# A Severe Case of Infantile Systemic Hyalinosis in an Asian Child: A Product of Consanguinity

**DOI:** 10.7759/cureus.16433

**Published:** 2021-07-16

**Authors:** Sumer Baroud, Ameen Alawadhi

**Affiliations:** 1 Department of Dermatology, Medical University of Sharjah, Sharjah, ARE; 2 Department of Dermatology, Salmaniya Medical Complex, Manama, BHR

**Keywords:** hyaline fibromatosis syndrome, infantile systemic hyalinosis, juvenile hyalinosis, fibromatosis, joint contractures, skin lesions

## Abstract

Infantile systemic hyalinosis (ISH) is a rare, autosomal recessive disorder characterized by widespread abnormal growth of hyalinized fibrous tissue in skin and mucosae. The typical clinical picture consists of the development of joint contractures, skin lesions, and severe, chronic pain. We report the case of a 2-year-old Pakistani girl, who presented to our clinic with papulonodular lesions, gingival hyperplasia, hypotonia, and joint contractures. Skin biopsy revealed hyaline deposits, and genetic testing revealed a mutation in the protein Anthrax toxin receptor 2 (ANTXR2).

## Introduction

Infantile systemic hyalinosis (ISH) is a very rare, systemic disorder that is inherited in an autosomal recessive fashion. It is characterized by the widespread deposition of hyaline material, similar to collagen type VI, in multiple organs [1,2]. Affected tissues and organs can include the dermis, skeletal and cardiac muscle, thyroid, spleen, gastrointestinal tract, and adrenal glands. The pathogenesis underlying the illness involves a mutation in the extracellular protein-binding domain of a protein called capillary morphogenesis protein-2 (CMG2), which is encoded by the Anthrax toxin receptor 2 gene (*ANTXR2*), as described by Hanks et al. and Deuquet et al. [3-5]. A mutation of the cytoplasmic domain of the same protein causes juvenile hyaline fibromatosis (JHF), a similar but milder disease.

The clinical features of ISH include painful swollen joint contractures, dermal abnormalities such as diminished skin elasticity, diffuse thickening with hyperpigmentation, and skin lesions such as pearly papules on the face and neck. Gingival hypertrophy and anal lesions have also been reported. Children with ISH frequently exhibit failure to thrive and increased susceptibility to both infections and bone fractures [6,7].

The diagnosis of ISH is made clinically; however, histological evidence of deposition of hyaline material in the skin or mucosae is confirmatory [8]. There is no specific treatment for ISH, with physical therapy and nutritional support being the only available means to improve the quality of life of patients. With that being said, many children with ISH die within the first two years of life, mostly due to recurrent respiratory infections and severe diarrhea [6].

We present a case of ISH with interest due to the consanguineous pedigree involved, and the severity of the presentation. By describing the spectrum of clinical features, we aim to facilitate early genetic diagnosis, counseling, and management.

## Case presentation

A 20-month-old Pakistani girl was referred to the dermatology clinic due to widespread skin lesions. Birth history showed that the patient was born at full term via normal spontaneous vaginal delivery following an uncomplicated and uneventful pregnancy. Weight at birth was 3.5 kg. During the first two months of life, the patient’s growth and development were routinely assessed at well-child visits and were appropriate for her age. She had been appropriately achieving developmental milestones. However, at the start of her third month of life, the patient’s mother started to notice impairment in active and passive limb movement. At this stage of workup, she was referred to the hospital's pediatric department and ultimately referred to the pediatric metabolic disease clinic.

The patient was admitted several times for recurrent infections, as well as for investigations regarding hypotonia. At the age of eight months, physical examination of the patient was notable for swelling on the lateral side of her nose and chin, as well as swelling of her gums. Subsequently, the patient developed cutaneous lesions on the face and scalp. During the following year, the patient's family failed to show up for follow-up visits.

**Figure 1:**
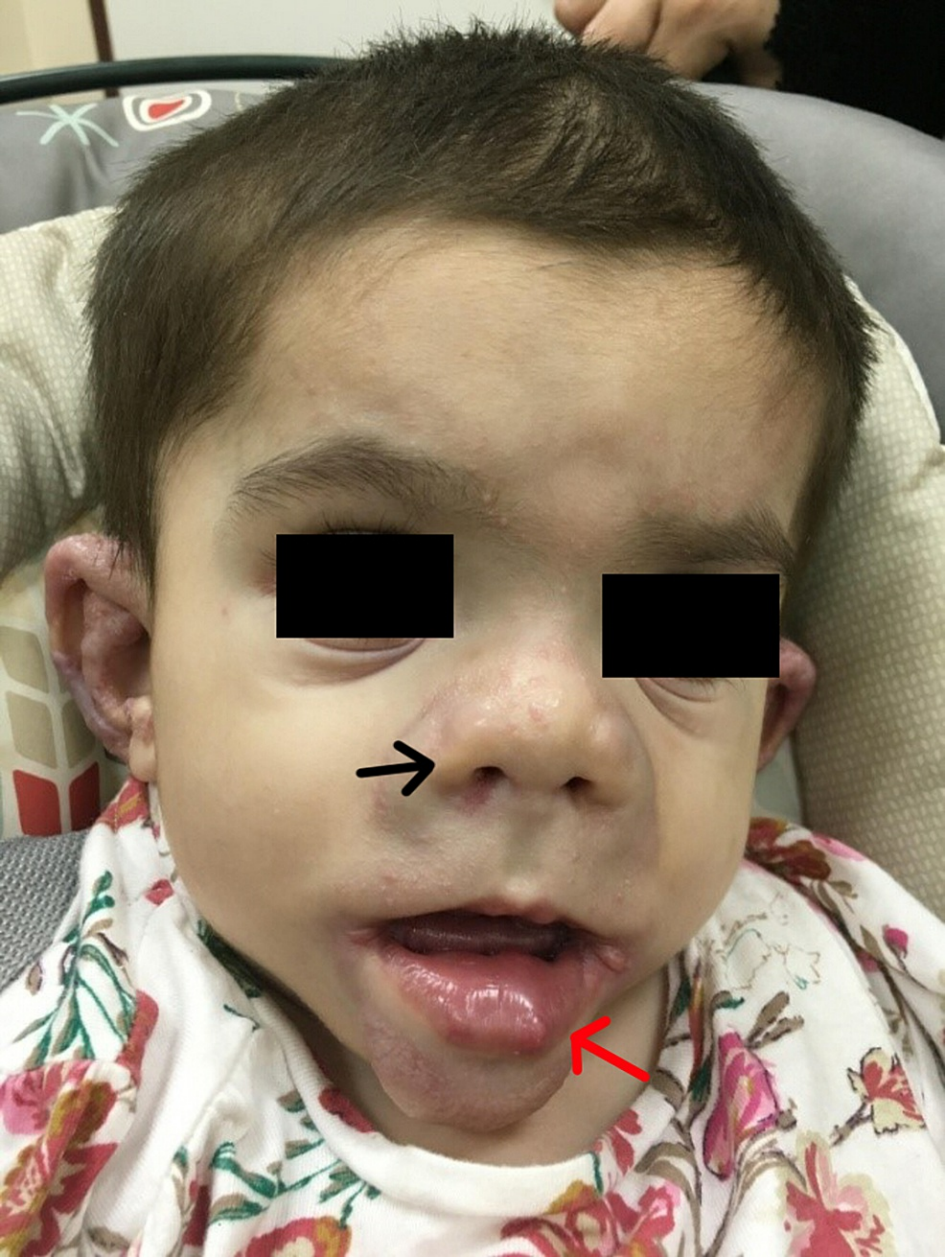
Saddle nose deformity with bilateral nodules around the nasal alae (black arrow) and swollen lips (red arrow)

**Figure 2:**
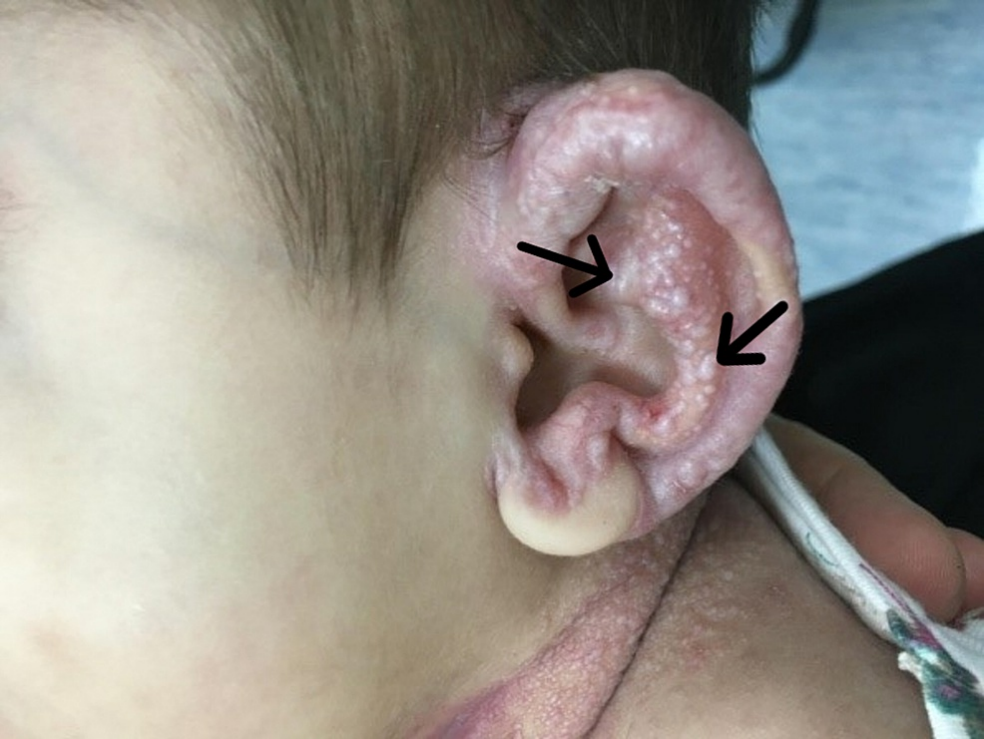
Multiple pearly pink and white papules coalescing into small plaques, mainly over the ears (black arrows)

**Figure 3:**
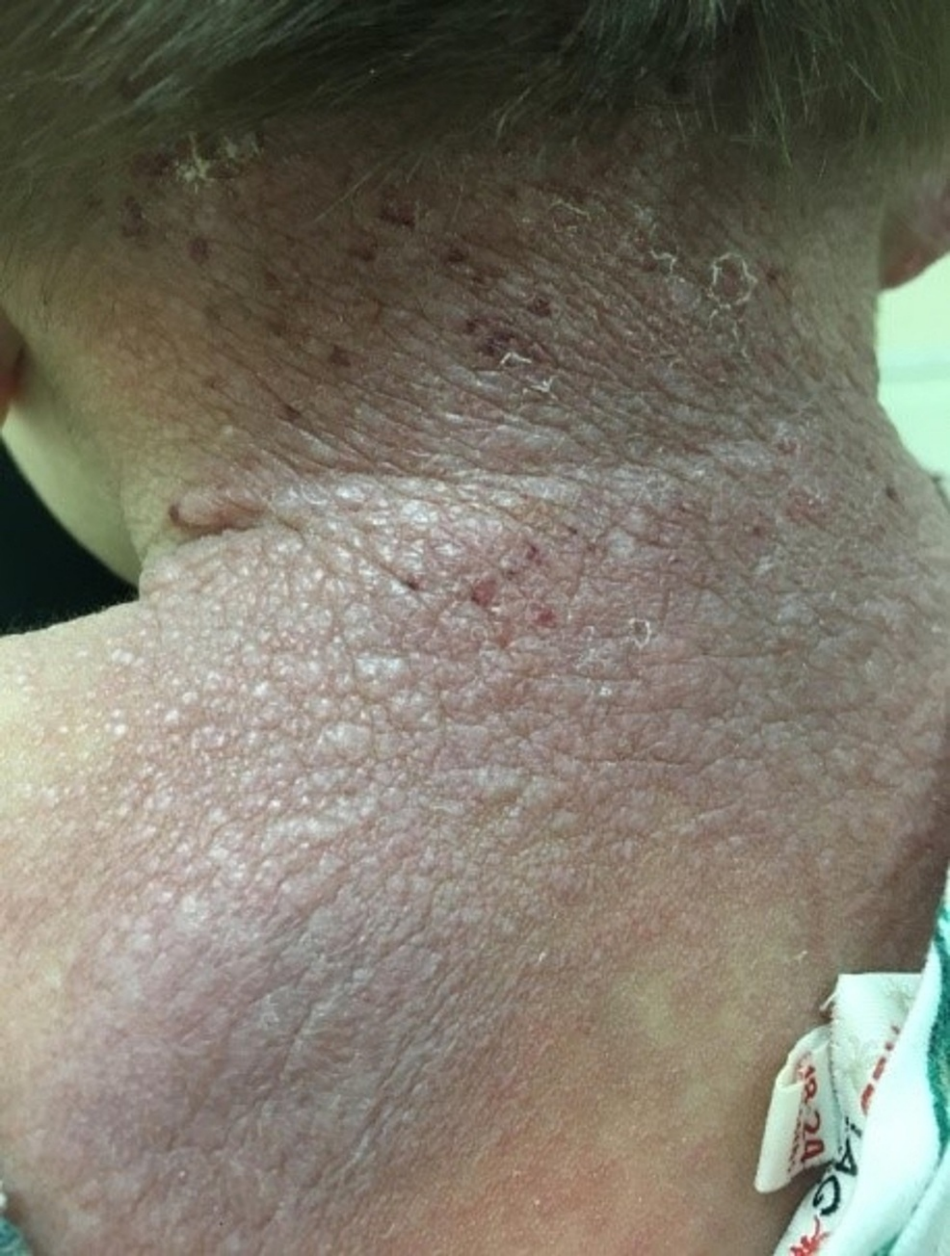
A thick, erythematous plaque over the upper back

**Figure 4:**
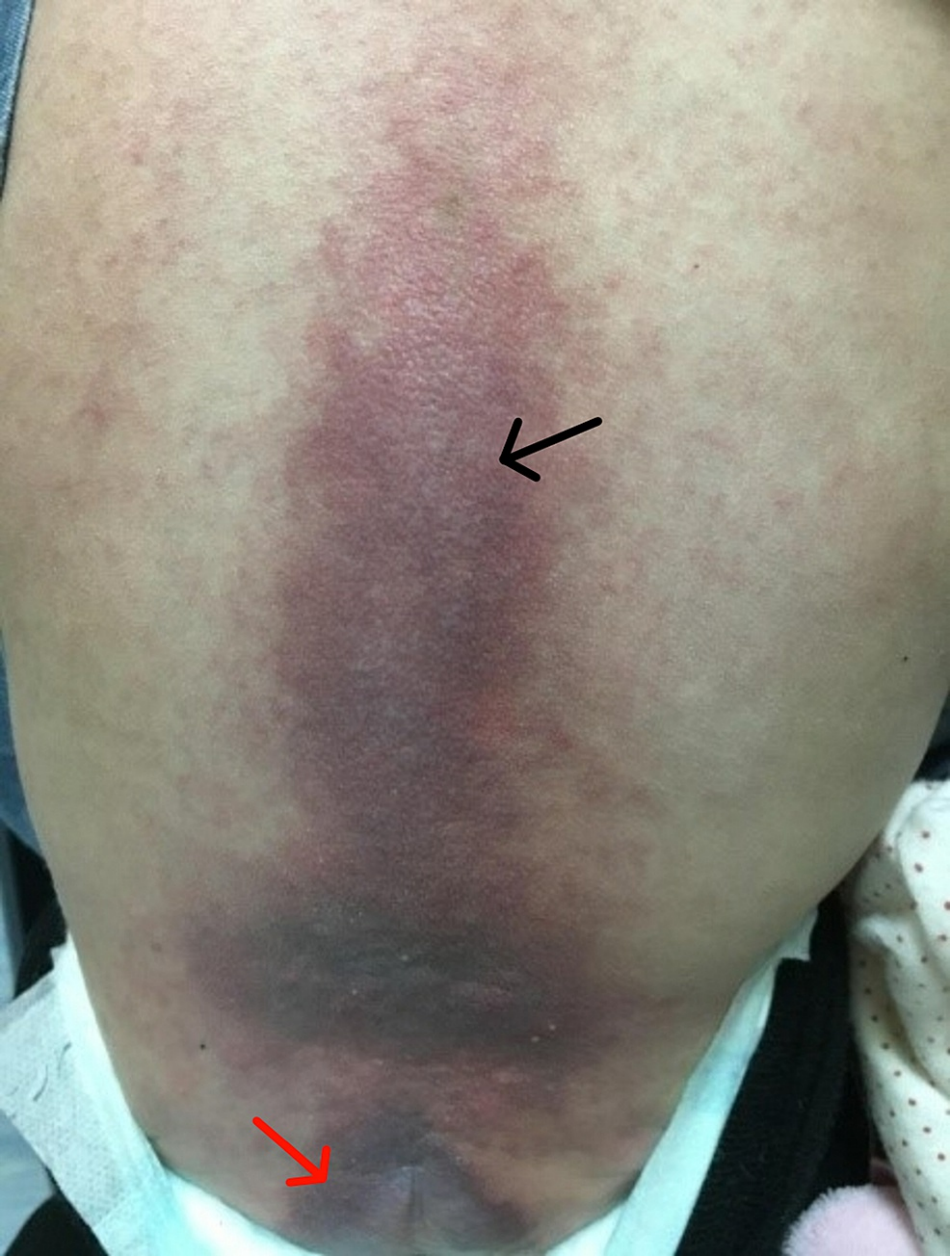
Well-demarcated, blanchable, violaceous patches over the back (black arrow) and buttocks (red arrow)

In March of 2017, the patient was brought to our dermatology clinic for her first visit. Physical examination demonstrated dysmorphic facial features, such as the presence of a saddle nose, bilateral nodules around the nasal alae, gingival hypertrophy, and macrocheilia (Figure 1). Cutaneous findings included multiple pearly pink and white papules over the face, ear, and neck, with some of the papules coalescing into small plaques, mainly over the ears (Figure 2). Other skin lesions included a thick, non-tender, longitudinal, erythematous plaque over the upper back (Figure 3), along with well-demarcated, blanchable, violaceous patches over the back and buttocks (Figure 4). Limb findings included hypotonia and joint stiffness.

**Figure 5:**
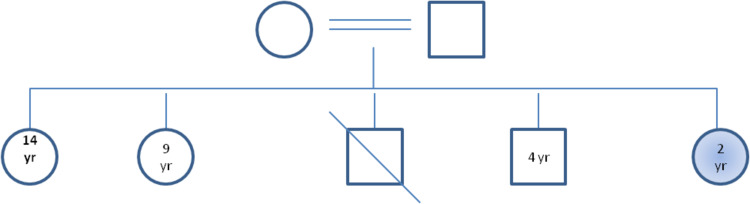
Family pedigree showing consanguinity

The patient is the youngest of five siblings, one of whom died at the age of eight months due to an unknown genetic disease that was diagnosed in Pakistan. The parents’ marriage is consanguineous, as displayed in the pedigree (Figure 5).

Differential diagnoses that were considered included ISH, JHF, Winchester syndrome, lipoid proteinosis (Urbach-Wiethe disease), and mucopolysaccharidosis type II (Hunter's syndrome). A punch biopsy of the nape of the neck was performed and sent for evaluation. Results showed a focally atrophic epidermis, with acellular eosinophilic interstitial hyaline material deposition in the dermis, favoring the diagnosis of ISH. Special staining for mucopolysaccharides was performed and turned out to be weakly positive, while special staining for amyloid turned out negative. Whole-genome sequencing was done and revealed a homozygous variant in the *ANTXR2* gene.

## Discussion

Infantile systemic hyalinosis (ISH) is an uncommon, systemic illness characterized by diffuse deposition of hyaline material in multiple organs and is thought to be inherited in an autosomal recessive pattern [6,9]. Due to this form of inheritance, this rare disease most commonly occurs in the offspring of consanguineous parents, a concept that is commonly practiced in the Middle East, West Asia, and North Africa. Affected patients display systemic findings starting from infancy. Affected tissues can include the skin, which may display subcutaneous nodules and pearly white to pink papules on the scalp, face, or ear. Other cutaneous findings that have been reported include perianal lesions, coarsened facial features, and hyperpigmented plaques [9]. Joint problems include chronic, painfully stiff joints and contractures that tend to reside with patients who manage to live to adolescence and adulthood, severely limiting mobility. Other affected organs may include skeletal muscle, cardiac muscle, thyroid, spleen, gastrointestinal tract, and adrenal glands, ultimately leading to multi-organ failure as a cause of death among patients with ISH. Other complications include chronic diarrhea and malnutrition as a result of protein-losing enteropathy [10]. Feeding problems that arise due to oropharyngeal deformities and hyaline deposition such as gingival hyperplasia, thickening of the oral mucosa, and peri-oral stiffness lead to failure to thrive, as well as an increased risk of infections, which often proves fatal in the first few years of life. The lack of appropriate nutrient intake ultimately leads to increased bone fragility and fractures [1,7,8,11,12].

A milder form of ISH, called JHF), presents with a similar clinical picture, although the prognosis of such patients is generally better. However, the patients that do survive until adulthood tend to suffer from crippling joint deformities [13].

The phenotypic variety associated with both ISH and JHF is due to one causative mutation: a mutation in a protein called CMG2, which is encoded by the *ANTXR2* gene [1,5]. CMG2 is a gene upregulated in endothelial cells to strongly bind to laminin and collagen IV, both of which are involved in capillary and basement membrane formation. A mutation in the extracellular protein-binding domain of CMG2 results in ISH, while in-frame and missense mutations within the novel cytoplasmic domain tend to result in the milder JHF [5].

Although the diagnosis of ISH is made clinically, it is important to confirm the disease histologically or at least rule out other possible differential diagnoses via appropriate testing. Histologic evaluation of cutaneous lesions and mucosal organs shows deposits of amorphous, fibrillar hyaline material, similar in appearance to type VI collagen [8,11]. Electron microscopy shows hyaline material deposited between the endothelial cells and pericytes, supporting the suggestion that ISH may result from leakage of plasma components through the basement membrane to the perivascular space [5].

Other differential diagnoses that should be ruled out before diagnosing ISH include JFH, lysosomal storage diseases, Winchester syndrome, lipoid proteinosis (Urbach-Wiethe disease), and mucopolysaccharidosis type II (Hunter’s syndrome). Clinically, ISH and JFH are often indistinguishable, with histological evaluation being similar in both diseases. This may support the suggestion that both diseases are part of a spectrum, rather than separate illnesses. However, failure to thrive, chronic diarrhea, and internal organ involvement are more common in ISH, hence the poorer prognosis [5,10,13].

There is no specific treatment for ISH. Physical therapy, pain control and nutritional support are the only available means to improve the quality of life of patients. Many children with ISH die within the first two years of life, mostly due to recurrent respiratory infections and severe malnutrition. Those who survive adolescence and adulthood suffer significant morbidity due to joint contractures and immobility. Patients with ISH are at risk of cardiomyopathy due to the hyaline deposition in cardiac muscle; therefore, cardiac evaluation may be advised as part of disease management [6,9].

## Conclusions

In summary, we present a severe case of ISH in a child born to consanguineous parents and review the illness. Along with gingival hyperplasia, hypotonia and joint contractures, cutaneous lesions such as papulonodular lesions are common manifestations of the disease. Skin biopsy often reveals hyaline deposits, and genetic testing confirms a mutation in *ANTXR2*. Due to the poor prognosis of the disease, genetic counseling should be given to parents, particularly those in consanguineous relationships, with children suffering from ISH, due to the risk of having future children with the same disease.
